# Disulfidptosis: a new form of regulated cell death for cancer treatment

**DOI:** 10.1186/s43556-023-00132-4

**Published:** 2023-06-15

**Authors:** Yu Meng, Xiang Chen, Guangtong Deng

**Affiliations:** Department of Dermatology, Xiangya Hospital, Central South University, Changsha, China

A recent study published in *Nature Cell Biology* demonstrated a novel type of regulated cell death (RCD) termed disulfidptosis that is caused by excessive disulfide formation in actin cytoskeleton and actin filament (F-actin) collapse, highlighting a promising anticancer strategy to target disulfidptosis [1].

As early as 2017, Gan et al. and his team found that SLC7A11 was overexpressed in several cancers, and SLC7A11 overexpression rendered tumor cells susceptible to glucose deprivation-triggered cell death [2]. In 2020, they further revealed that SLC7A11-mediated cystine uptake and subsequent cystine reduction to cysteine induces intracellular disulfide accumulation, NADPH depletion and ultimate cell death under glucose starvation [3]. As with ferroptosis, this cell death is accompanied by depletion of GSH and NADPH, whereas cystine starvation promotes ferroptosis yet inhibits this cell death, indicating a previously unrecognized type of cell death. Moreover, SLC7A11 overexpression is well-known to prevent ferroptosis via increased GSH levels and attenuate necroptosis via GPX4-mediated chelation of lipid peroxidation [4]. It seems that SLC7A11 inhibits ferroptosis and necroptosis at the cost of promoting glucose starvation-induced cell death. However, a well-acknowledge theory and precise mechanism underlying this form of cell death still need to be further clarified.

In this study, Gan et al. firstly investigated whether the glucose starvation-induced cell death in SLC7A11-overexpressing cancer cells was dependent on known cell death pathways. Pharmacological or genetic inhibition of apoptosis (using the caspase inhibitor Z-VAD-FMK or double knockout BAX/BAK), ferroptosis (using ferrostatin-1 or deferoxamine or knockout ACSL4), necroptosis (using necrostatin-1/2), and autophagic cell death (using chloroquine) did not exert any rescuing effect on the cell death. Additionally, elevated ATP levels, no cystine crystal formation, and inability of reactive oxygen species (ROS) scavengers to rescue the cell death ruled out the possibility of cell death associated with ATP depletion, crystal damage, and ROS accumulation. In contrast, disulfide-reducing agents including dithiothreitol, β-mercaptoethanol (2-ME), and tris-(2-carboxyethyl)-phosphine, almost completely suppressed the cell death. Therefore, this form of cell death differs from the known cell death patterns and is regulated by disulfide-reducing agents, hereafter called disulfidptosis due to disulfide stress.

To determine the specific target proteins for disulfidptosis, authors applied the bio-orthogonal chemical proteomic approach and found that glucose deprivation induced disulfide formation in actin cytoskeleton in SLC7A11-overexpressing cells. Notably, glucose deprivation caused NADPH depletion at 1 h, and slowed down the migration of actin cytoskeleton proteins at 2 h, and ultimately led to disulfidptosis. Inhibiting NADPH depletion with cystine starvation, increasing NADPH supply with 2-deoxyglucose (2-DG), or treatment with disulfide-reducing agent 2-ME prevented the retarded migration of these actin cytoskeleton-associated proteins and disulfidptosis. Therefore, glucose starvation-induced NADPH depletion is more likely to cause disulfide formation in actin cytoskeleton and subsequent disulfidptosis. However, it is unclear whether NADPH depletion selectively triggers the excessive disulfide formation in actin cytoskeleton, and it is urgent to explore whether other sulfhydryl-containing proteins are influenced similarly during disulfidptosis.

To further explore the actin cytoskeleton dynamics during disulfidptosis, authors performed phalloidin staining for F-actin, and found cell shrinkage and F-action contraction and separation from the cell membrane. Notably, the morphological change preceded disulfidptosis and could be suppressed by treatment with cystine starvation, 2-DG and 2-ME, suggesting that excessive disulfide formation in the actin cytoskeleton caused F-actin contraction and collapse during disulfidptosis. Authors further performed a genome-wide CRISPR/Cas9 lose-of-function screen analysis. Among suppressor hits, NCKAP1, a component of WAVE regulatory complex (WRC), stood out for its roles in promoting actin polymerization and lamellipodia formation. Deletion of NCKAP1 or other components in the WRC partially suppressed disulfidptosis, while overexpression of active form Rac1, the activator of WRC, promoted lamellipodia formation and disulfidptosis. These results suggest that Rac1-WRC-mediated lamellipodia formation contributes to disulfidptosis, further highlighting disulfidptosis as a novel form of RCD. However, it is unclear why lamellipodia formation is necessary for disulfide crosslinking among actin cytoskeleton and disulfidptosis, and multi-omics study is needed to determine whether pathways and targets other than actin cytoskeleton proteins play a more significant role in disulfidptosis.

Finally, for further verification of the mechanism of disulfidptosis, pharmacological inhibition of glucose uptake with glucose transporter (GLUT) inhibitors were used in SLC7A11-overexpressing tumor cells, and the results demonstrated the same cytotoxicity as glucose starvation, accompanied by excessive disulfide formation in actin cytoskeleton and F-actin collapse. Likewise, the GLUT inhibitor BAY-876 inhibited tumor growth and triggered disulfide bonds in actin in PDX tumors with SLC7A11 overexpression, but had no effect in PDX tumors with SLC7A11 low expression. The supplementation of cystine also induced disulfidptosis in cancer cells with SLC7A11 low expression, accompanied by intracellular NADPH depletion, aberrant disulfide frormation in actin cytoskeleton and F-actin contraction. Thus, disulfidptosis could be induced in cancer cells regardless of SLC7A11 expression, and targeting disulfidptosis with glucose inhibitors offers new potential for cancer treatment. However, whether disulfidptosis occurs under other metabolic-stress conditions accompanied by NADPH depletion and its physiological meaning needs further investigations.

**Fig. 1:**
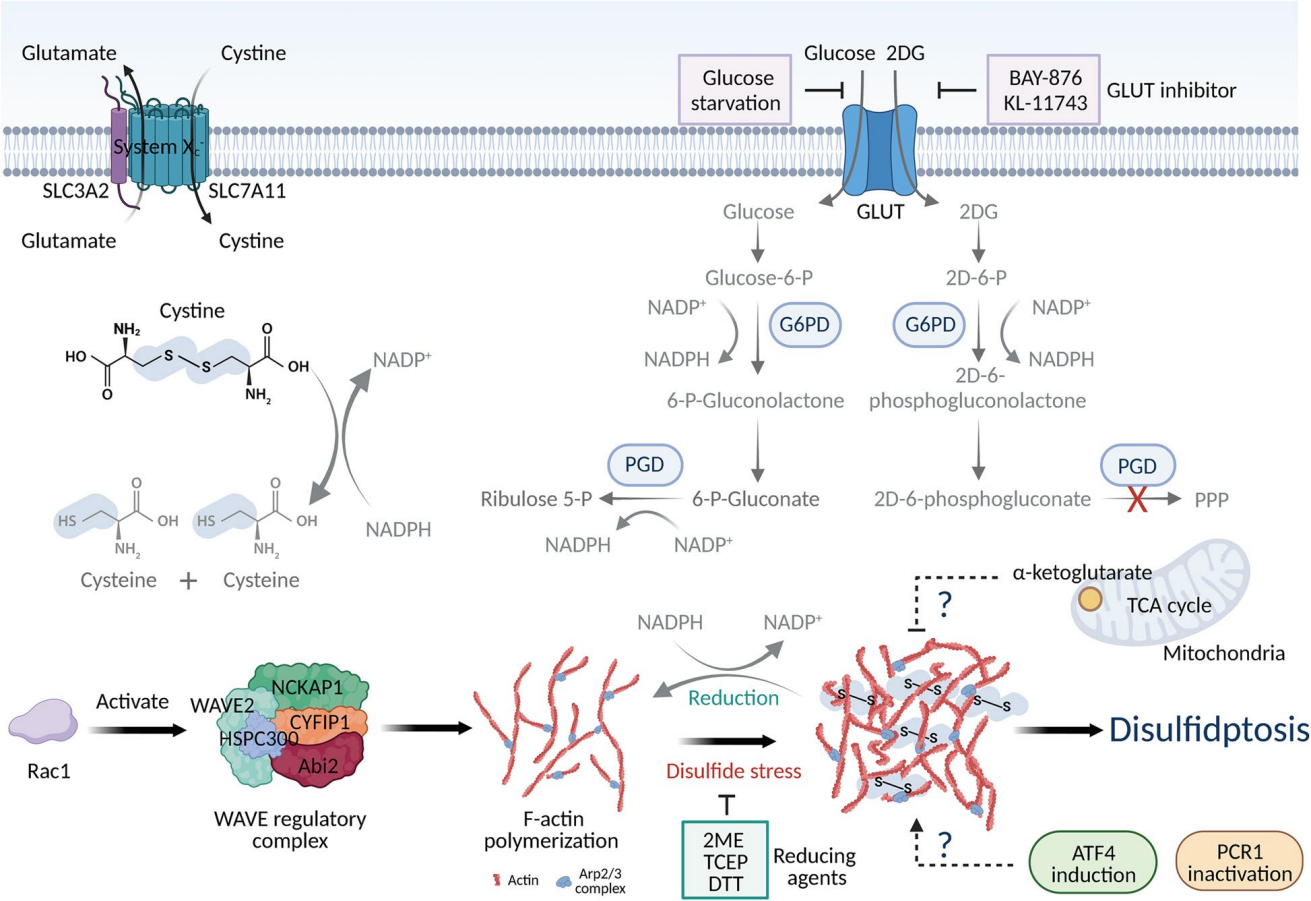
Schematic model of disulfidptosis mechanism. When NAPDH production is blocked from the pentose phosphate pathway by glucose starvation or GLUT inhibitors (BAY-876, or KL-11743), abundant intracellular cystine imported through the system X_c_^−^ would deplete cellular NADPH, resulting in large amounts of cystine accumulation, aberrant disulfide-bond formation in actin cytoskeleton proteins and F-actin collapse, ultimately resulting in disulfidptosis. G6PD, 6-phosphoglucose dehydrogenase; PDG, 6-phosphogluconate dehydrogenase; TCA, tricarboxylic acid cycle; 2-DG, 2-deoxyglucose; GLUT, glucose transporter; 2-ME, β-mercaptoethanol; TCEP, tris-(2-carboxyethyl)-phosphine; DTT, dithiothreitol; Rac1, rac family small GTPase 1; ATF4, activating transcription factor 4; PCR1, polycomb-repressive complex 1

In conclusion, this study not only reveals a previously unidentified form of RCD, but also provides an in-depth analysis of the mechanism of disulfidptosis (Fig. 1). Authors previously suggested that supplementation of α-ketoglutarate could rescue disulfidptosis [2]. The conversion of α-ketoglutarate to succinyl-CoA with the generation of NADH, another reducing agent, provides a potential explanation for the recused effect. Moreover, PRC1 inactivation promoted but ATF4 deletion inhibited disulfidptosis, probably due to their role in endoplasmic reticulum stress, which is responsible for the formation of disulfide bonds in proteins [5]. However, the exact underlying mechanisms still need further exploration. Like any ground-breaking research, such an ingenious study facilitates multiple interesting questions in the future. What are the thresholds of disulfide-bond formation in actin cytoskeleton proteins required to induce disulfidptosis? How is disulfidptosis initiated, propagated, and finally executed in cancer cells? What is the role of mitochondria in disulfidptosis, by virtue of its essential roles in other forms of RCD? Whether could the susceptibility of disulfidptosis be used for selecting appropriate cancer patients for GLUT inhibitors in future preclinical and clinical studies? Whether metastatic cancer cells are more sensitive to disulfidptosis for their enhanced lamellipodia?
